# Overcoming the probing-depth dilemma in spectroscopic analyses of batteries with muon-induced X-ray emission (MIXE)†

**DOI:** 10.1039/d4ta05112b

**Published:** 2024-12-04

**Authors:** Edouard Quérel, Sayani Biswas, Michael W. Heiss, Lars Gerchow, Qing Wang, Ryo Asakura, Gian Müller, Debarchan Das, Zurab Guguchia, Fabian Hotz, Gianluca Janka, Andreas Knecht, Hubertus Luetkens, Charles Mielke, Carlos Vigo, Chennan Wang, Stergiani Marina Vogiatzi, Toni Shiroka, Thomas Prokscha, Katharina von Schoeler, Shunsuke Asari, I.-Huan Chiu, Akira Sato, Kazuhiko Ninomiya, Megumi Niikura, Corsin Battaglia, Alex Amato, Arndt Remhof

**Affiliations:** a Empa, Swiss Federal Laboratories for Materials Science and Technology Dübendorf 8600 Switzerland edouard.querel@empa.ch; b Center for Neutron and Muon Sciences, Paul Scherrer Institute 5232 Villigen PSI Switzerland sayani.biswas@stfc.ac.uk; c Institute for Particle Physics and Astrophysics, ETH Zürich Zürich 8093 Switzerland; d Graduate School of Science, Osaka University 1-1 Machikaneyama Toyonaka 560-0043 Osaka Japan; e Institute of Radiation Sciences, Osaka University Toyonaka Osaka Japan; f RIKEN Nishina Center for Accelerator-Based Science 2-1 Hirosawa Wako 351-0198 Saitama Japan; g Department of Information Technology and Electrical Engineering, ETH Zürich 8092 Zürich Switzerland; h Institute of Materials, School of Engineering, EPFL Lausanne 1015 Switzerland

## Abstract

Battery research often encounters the challenge of determining chemical information, such as composition and elemental oxidation states, of a layer buried within a cell stack in a non-destructive manner. Spectroscopic techniques based on X-ray emission or absorption are well-suited and commonly employed to reveal this information. However, the attenuation of X-rays as they travel through matter creates a challenge when trying to analyze layers buried at depths exceeding hundred micrometers from the sample's surface. In the context of battery research, the limited escape depth of X-rays often necessitates the design of experiment-specific cells with thinner inner layers, despite the risk that these tailored cells may not exactly replicate the cycling behavior of larger commercial cells. Muon-induced X-ray emission (MIXE) is a non-destructive spectroscopic technique that involves implanting negative muons into a sample and detecting the highly energetic muonic X-rays generated when these muons are captured by the sample's atoms. By virtue of the high energy of muonic X-rays, the depth of analysis of MIXE greatly exceeds that of other X-ray based techniques. In this article, we introduce the technique and lay the groundwork for employing MIXE in future *in situ*/*operando* analyses of batteries. We demonstrate that MIXE can detect nearly all elements, including low atomic number ones such as Li. Additionally, we establish the quantitative nature of MIXE through the precise determination of LiNi_*x*_Mn_*y*_Co_1−*x*−*y*_O_2_ (NMC) electrode stoichiometries. Finally, we demonstrate that MIXE enables the acquisition of depth-resolved chemical information from a 700 μm thick cell, in good agreement with simulation results.

## Abstract 2

An introductory guide to muon-induced X-ray emission (MIXE) for the battery community, highlighting both the potential and limitations of this novel characterization technique with extended probing depth.

## Graphical abstract

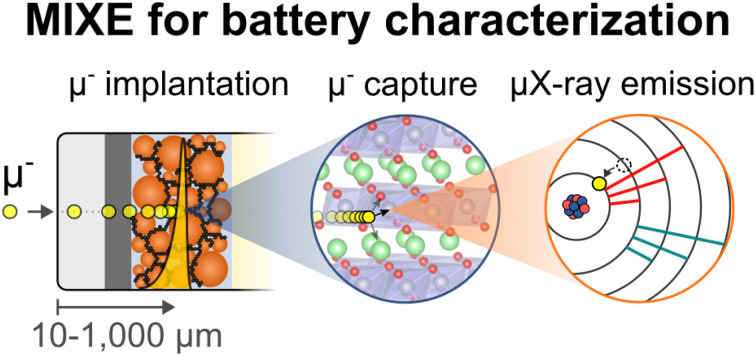

## 1 Introduction

Developing techniques to analyze (electro)chemical reactions as they occur inside a battery without having to disassemble the cell is a valuable approach for understanding the performance and degradation of battery materials. Electrochemical data obtained *via* (dis-)charging cells offer some insights, but often do not provide a complete picture of the underlying chemical and physical phenomena affecting cell behavior. Hence, additional material characterization techniques that can investigate these reactions in a non-destructive manner, either *in situ* or *operando*, are needed. *In situ* experiments have particular advantages over analyses performed on disassembled cells as they prevent alteration of air-sensitive cell constituents when exposed to the atmosphere, thereby ensuring the accuracy of conclusions drawn regarding reactions inside the cell. Beyond *in situ* measurements, *operando* measurements conducted during cell cycling offer the additional advantage of capturing the onset and kinetics of reactions.

Spectroscopic techniques that rely on the detection of X-rays emitted by a sample play a crucial role in battery research. They help determine the elemental composition and chemical state of a sample. While some techniques provide spatially averaged chemical information over a probing volume, more detailed insights into cells can be obtained through spatially resolved techniques. These techniques can be depth-resolved (1D), surface-resolved (2D) or tomographic (3D). Established spatially resolved X-ray techniques used in battery studies include electron dispersive X-ray spectroscopy (EDX/EDS), X-ray fluorescence spectroscopy (XRF), or X-ray absorption spectroscopy (XAS) and its derivatives such as computed tomography combined with X-ray absorption near-edge structure spectroscopy (CT-XANES).^1^ One limitation shared by X-ray based techniques is their probing depth, which is defined by the absorption of X-rays in matter that rarely exceeds 100 μm. This limitation affects the types of batteries that can be analyzed, often requiring specific cell holders and customized cell thicknesses. A battery cell in an industry-standard pouch cell format can typically not be analyzed, because any signal from the cell would hardly escape the ∼100 μm thick pouch foil consisting of a stacked polyamide, aluminum, and polypropylene layer. Hence, a method offering a more extended probing depth would be beneficial to conduct spectroscopic analyses on research and industrial battery cells.

Muonic X-rays, emitted when negative muons hit a sample and are captured by its atoms, have energy levels approximately two orders of magnitude greater than electronic X-rays. These higher energies, in the range of keV to several MeV, allow muonic X-rays to travel further before being absorbed by matter. In the 1970s and 1980s, researchers employed muonic X-ray emission for material characterization for the first time.^2–6^ The technique, known as muon induced X-ray emission (MIXE)§§The technique is not uniformly named in the literature and is sometimes also called Muonic X-ray Emission Spectroscopy (μXES) or Muonic X-ray Analysis (MXA)., was not extensively explored for several decades due to the low intensity of negative muon beams. This resulted in long acquisition times and relatively poor signal-to-noise ratios. However, with the availability of high intensity muon beams, MIXE is now experiencing a revival, enabling shorter experimental durations. MIXE is a non-destructive technique that provides depth-resolved X-ray spectroscopy with a probing depth in the order of 10–10 000 μm, surpassing the capabilities of electronic X-ray techniques. MIXE therefore holds great potential to overcome probing depth limitations in spectroscopic analyses of batteries. Recently, a few studies have started to investigate the applicability of the technique to study Li-ion batteries.^7,8^ Compared to neutron-based techniques, which offer unmatched depth profiling capabilities, MIXE uniquely provides direct elemental composition at each probing depth from the measured spectrum, applicable to both amorphous and crystalline phases, without requiring refinement or prior knowledge of the crystal structure.

This article serves as an introductory guide to MIXE for the battery community, highlighting its potentials and limitations. Starting with an overview of MIXE's fundamental principles, the article then delves into its advantages for battery analysis. Our study shows that MIXE is capable of detecting nearly all elements, including lithium under certain conditions. The quantitative nature of MIXE is also confirmed by measuring the elemental ratios of three LiNi_*x*_Mn_*y*_Co_1−*x*−*y*_O_2_ (NMC) positive electrodes with different Ni : Mn : Co ratios. The extended depth-profiling capability of MIXE is demonstrated by distinguishing the elemental composition of the different layers of a single stack pouch cell with a thickness of ∼700 μm. Our results show that MIXE can differentiate isotopes and our studies are ongoing to differentiate oxidation states of elements. Finally, while this article only includes experiments on Li-ion batteries, the applicability of MIXE spans a broader spectrum of battery technologies, including, among others, Na-ion, lithium–sulfur, or solid-state batteries.

## 2 Fundamental principles of muon induced X-ray emission (MIXE)

### 2.1 Muon production

Muons, like electrons, are elementary particles with a spin of 1/2, belonging to the class of leptons, with a mass that is ∼207 times that of electrons. They can be produced at proton accelerator facilities, such as the High Intensity Proton Accelerator complex at the Paul Scherrer Institute (PSI).^9,10^ In the first step, the proton beam is directed to a carbon target to generate pions (π^+^ and π^−^). These pions decay into both positive and negative muons (μ^+^ and μ^−^), from which the negative muons are selected for MIXE. These muons are then guided through a series of beam optics elements to (1) reduce contamination of other particle species, (2) retain μ^−^ of a given momentum (*p*) within a certain momentum bite (Δ*p*/*p*), and (3) shape the beam, depending on the requirements.

### 2.2 MIXE experiment

**Fig. 1:**
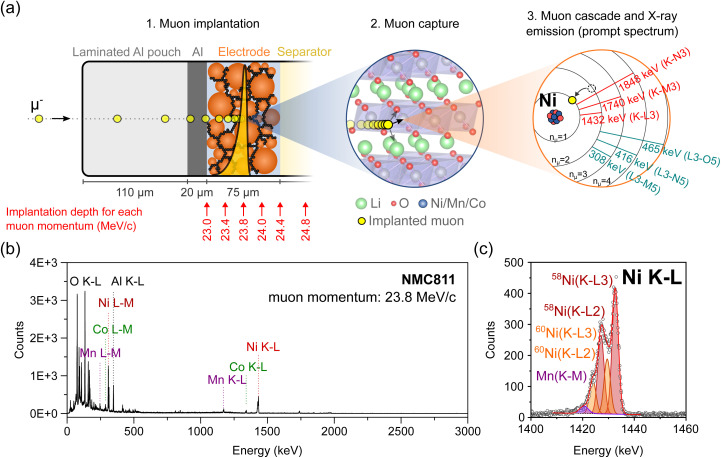
(a) Schematics illustrating the successive steps leading to muon-induced X-ray emission (not to scale): (1) a muon with a selected momentum (here 23.8 MeV/c) is implanted at a defined depth inside the pouch cell; the yellow dots represent the time lapse of a single muon stopping in the battery; the yellow Bragg curve represents the stopping profile of muons with a defined momentum; (2) the muon is then captured within the Coulomb field of a neighboring nucleus (the crystalline structure of an NMC electrode is depicted here); (3) the muon rapidly cascades down to the innermost (*n* = 1) shell, thereby inducing characteristic X-ray emission. (b) Full-range MIXE spectrum collected in 30 minutes from a NMC811‖graphite pouch cell where the muons were implanted in the NMC electrode at a momentum of 23.8 MeV/c; the positions of a few muonic X-rays of interest are indicated; (c) magnification and fitting of the Ni K–L region.

During an experiment, such as the one represented in Fig. 1(a), the beam of μ^−^ with momenta typically in the range of 20–30 MeV/c is directed at the battery cell, which is typically mounted on a frame, in air, at a distance of ∼10 cm from the muon counter through which the μ^−^ exit the beamline (photographs of the experimental setup are presented in Fig. S1†).^11^ As they penetrate matter, negative muons experience a deceleration resulting from electrostatic interaction with the outer electrons (and to a lower degree the nuclei) of atoms in their paths. The average penetration depth of muons mainly depends on their initial momentum *p*, the density *ρ*, and porosity of the different layers of matter that the muons travel through. The stopping profile of μ^−^ can be modelled for different materials with simulation tools, such as the Particle and Heavy Ion Transport code System (PHITS 2.88) or the Geant4 toolkit (v11.1.1).^12–14^ When the thickness and composition of the different layers of a sample are already known, like for our Li-ion batteries, these simulations are used prior to conducting the experiment to determine the momentum at which incoming muons should be implanted in the sample for them to stop at a desired depth. With our current setup, muon momenta are typically tuned in a range of 15–60 MeV/c.¶¶This range is mainly given by technical limitations of the employed beamline and the sample environment (air) and can be extended by appropriate modifications, if required. This range of momenta allows muons to be implanted in the layers of a battery at depths exceeding a millimeter from the surface. The deceleration process is a statistical one resulting in the distribution of implantation depths (straggling) even in the case of an ideal monoenergetic muon beam. The straggling depends on the muon momentum and the density and heterogeneity of the target material. In addition, it will be enhanced by the momentum bite (Δ*p*/*p*, which is the tolerated deviation from nominal momentum, typically set to 0.5% or 1%) of the beamline.

Once stopped, μ^−^ are captured in the Coulomb field of a nearby nucleus. The resulting muonic atom is typically created in an excited state, with the muon in a muonic orbit with principal quantum number *n*_μ_ ∼ 14.^15,16^ Subsequently, the muon relaxes in a time-scale in the order of 10^−13^ s to the lowest *n*_μ_ = 1 muonic orbit by emitting a series of muonic X-rays (μ-X rays). Similarly to electronic X-rays, the energy of μ-X rays is element specific. The μ^−^ thereby act as non-destructive local probes of the chemical environment at their implantation depth. Depth-resolved information about the composition, isotopes, and in certain cases, oxidation state of the elements can be achieved by collecting muonic X-ray spectra, such as the ones depicted in Fig. 1(b) and (c), at various μ^−^ implantation depths.

One decay mechanism for muonic atoms is through the capture of the μ^−^ by the nucleus, which is then in an excited state and mostly decays *via* gamma-ray emission.

The five muon beam lines around the world (the SμS at the PSI in Switzerland, ISIS at the RAL in the UK, MUSE at J-PARC in Japan, MuSIC at RCNP in Japan, and TRIUMF in Canada) can be categorized by their muon production modes: pulsed or continuous. Previous work by our group demonstrated that continuous muon sources, such as the one at the PSI, are highly advantageous for MIXE because muons can be implanted one at a time with an average of >10 muon lifetimes between two implantations, thereby limiting the risk of pile-up events in the high-purity germanium detectors used to collect the muonic X-rays and thus determining more accurately the X-ray energies.^17^ Since 2020, our team has worked on the development of GIANT,^11^ an advanced X-ray detection system specifically adapted to MIXE experiments (see Fig. S1†), which makes our MIXE setup at the PSI a world-leading platform for the depth-resolved elemental analysis of objects that cannot be studied by destructive analysis techniques including precious archaeological artefacts and meteorites,^18,19^ or operating devices such as batteries.

### 2.3 MIXE for battery characterization

The probing depth of MIXE provides a distinctive advantage over other X-ray emission and absorption techniques for characterizing batteries in commercial cell formats (*e.g.* pouch cells) without requiring customization of the cells to accommodate experimental limitations.

MIXE is also uniquely positioned to study long-range elemental composition changes occurring during battery cycling. Long-range ion transport occurs during every (dis)charge cycle of a battery, including when (1) the charge carrier ions, *e.g.* Li^+^ ions for Li-ion batteries (LIBs), are reversibly exchanged between the positive and negative electrodes of a cell; (2) to a lesser extent, parasitic ions originating from the dissolution of the current collectors or electrodes become mobile under certain operating conditions and deposit as undesired ectopic layers, *e.g.* by plating on the counter electrode.^20^

Developing an *operando* technique which can be used to probe the charge carrier concentration as a function of depth would be extremely useful to understand concentration gradients arising from limitations in (dis)charge kinetics across the thickness of electrodes. This characterization need becomes even more urgent to solve as the trend is to make electrodes thicker to increase the cell-level energy density.^21^ If the same technique can also be used to understand which cycling conditions promote the dissolution of parasitic ions, including transition metals from the positive electrodes or current collector atoms, this could offer a platform for researchers to improve the longevity and performance of cells.

A recent study also demonstrated that MIXE can be used to detect the plating of metallic lithium on overcharged graphite electrodes;^8^ this undesired reaction could be studied *operando via* MIXE to capture the onset of metallic lithium plating under various experimental conditions (*e.g.* temperature and charging speed). This also has potential for the study of metallic lithium filament short-circuits in solid-state batteries.^22^

## 3 Materials and methods

### 3.1 Reference materials

The detectability of lithium by MIXE was tested by measuring the signal of pure Li metal foil (China Energy Lithium Co. Ltd, 200 μm thick) in a vacuum-sealed pouch (MTI, 115 μm total thickness, consisting of 25 μm polyamide, a 40 μm aluminum foil layer and 40 μm polypropylene layers held together by ∼10 μm polyester–polyurethane adhesives) and comparing it to the signal of an empty pouch.

Pieces of NMC111 (Customcells, 2 mA h cm^−2^, 41 μm thick porous electrode on 20 μm thick Al foil), NMC622 (Customcells, 2 mA h cm^−2^, 39 μm thick porous electrode on 20 μm thick Al foil, 38% porosity), and NMC811 (Customcells, 3.5 mA h cm^−2^, 75 μm thick porous electrode on 20 μm thick Al foil, 39% porosity) single-side coated electrode foil were vacuum-sealed into laminated Al pouches and analysed to evaluate the feasibility of detecting Li present in the cathode and confirm the quantitative nature of MIXE by calculating the transition metal ratios.

### 3.2 Pouch cell preparation

A NMC811‖graphite pouch cell was assembled from a commercial single-side coated NMC811 electrode (Customcells, 3.5 mA h cm^−2^, 75 μm thick on 20 μm thick Al foil, 3 × 4 cm^2^ area, 39% porosity) and a graphite electrode (Customcells, 4.1 mA h cm^−2^, 3.5 × 4 cm^2^ area, 86 μm thick, 37% porosity) as positive and negative electrodes, respectively, resulting in an n : p ratio of 1.37. The NMC electrode consisted of 94.5 wt% NMC811 active particles, with the rest being the polyvinylidene fluoride (PVDF) binder and carbon black conductive additive. The active material fraction in the graphite electrode was 95 wt%, with the rest being carboxymethyl cellulose (CMC) and styrene–butadiene rubber (SBR) as binders.

Battery tabs (MTI, Ni for the graphite electrode, and Al for the NMC electrode) were spot welded to the current collectors. All electrodes were dried under vacuum at 120 °C for 12 h (Büchi B585 oven) and transferred to an Ar-filled glovebox (MBraun, O_2_ < 1 ppm, H_2_O < 1 ppm) for assembly.

The pouch cells were assembled inside the glovebox using a borosilicate glass fiber separator (Whatman GF/A, with a thickness of 260 μm, facing the NMC electrode) in combination with a polypropylene separator (Celgard 2500, 25 μm thick, facing the graphite electrode). The electrolyte for the cells was 1200 μL of 1 M LiPF_6_ in ethyl carbonate : ethylene carbonate (EC : EMC), 3 : 7 by volume (Solvionic) with a 2 wt% addition of vinylene carbonate (VC, E-Lyte). The cells were vacuum-sealed into laminated Al pouches.

### 3.3 MIXE simulations

As shown in Fig. 4, simulations of muon implantation were conducted using Geant4 and PHITS.^12–14^ The simulations were carried out from *p* = 18.0 to 30.0 MeV/c, at intervals of 0.1 MeV/c. The 200 μm-thick muon-entrance counter, the 10 μm-thick Ti window, and the 10 cm of air that negative muons traverse before hitting the sample were included in the simulations. The simulations were conducted for a momentum spread of Δ*p*/*p* = 0.5%.

All the parameters used for the simulations are included in Table S1 in the ESI File.†

### 3.4 MIXE measurements

The MIXE data were collected at the πE1 beamline at the Swiss Muon Source of the High Intensity Proton Accelerator complex at the PSI. All details about the detection setup and data analysis protocols can be found in one of our previous publications.^11^

The X-ray energies for the element identification are taken from the theoretical calculations of mudirac.^23^ In all the figures, the muonic X-ray peaks are labelled by their respective element and transition following the IUPAC notation. The fitting algorithms implemented in CasaXPS were used to analyze the MIXE spectra.^24^ A Gaussian lineshape was employed to fit individual peaks. As shown in Fig. 4(c), the normalized intensities for muonic X-rays were obtained by dividing the measured intensities of muonic X-rays by (1) the total muon implantation events in that spectrum and (2) the combined efficiency of the High Purity Germanium (HPGe) detectors at these energies.

The energy and efficiency calibrations of the HPGe detectors were performed using standard radioactive gamma-emitting sources with known activities and reference dates. The following radioactive sources were used (with a 3% error in the activities from the manufacturer): ^88^Y, ^152^Eu, ^241^Am, ^210^Pb, ^60^Co, ^133^Ba, ^57^Co, and ^109^Cd. The absolute efficiency could be fitted to a quadratic polynomial function:

1 ln(*η*) = *a*_0_ + *a*_1_ ln(*E*/1000) + *a*_2_(ln(*E*/1000))^2^

where *E* is the muonic X-ray energy and *a*_0_, *a*_1_, and *a*_2_ are the fitting parameters. With the detection setup used to measure the data presented in Fig. 4, the obtained values of the fitting parameters were *a*_0_ = 1.27257 × 10^−1^, *a*_1_ = −8.90527 × 10^−1^, and *a*_2_ = −8.76841 × 10^−2^.

## 4 Results and discussion

### 4.1 Lithium detectability

Lightweight elements such as lithium exhibit low-energy electronic X-ray transitions due to the relatively weak binding energy of the core electrons. With photon energies approximately around 54 eV, the Li K–L emission band falls within the ultra-soft X-ray range. This makes lithium particularly hard to detect *via* X-ray spectroscopic techniques such as EDX or XRF because such low energy X-rays are easily absorbed within the sample and cannot escape distances exceeding 10–100 nm. Light element analysis is therefore impossible if the surface of the sample is not directly exposed to the detectors.

**Fig. 2:**
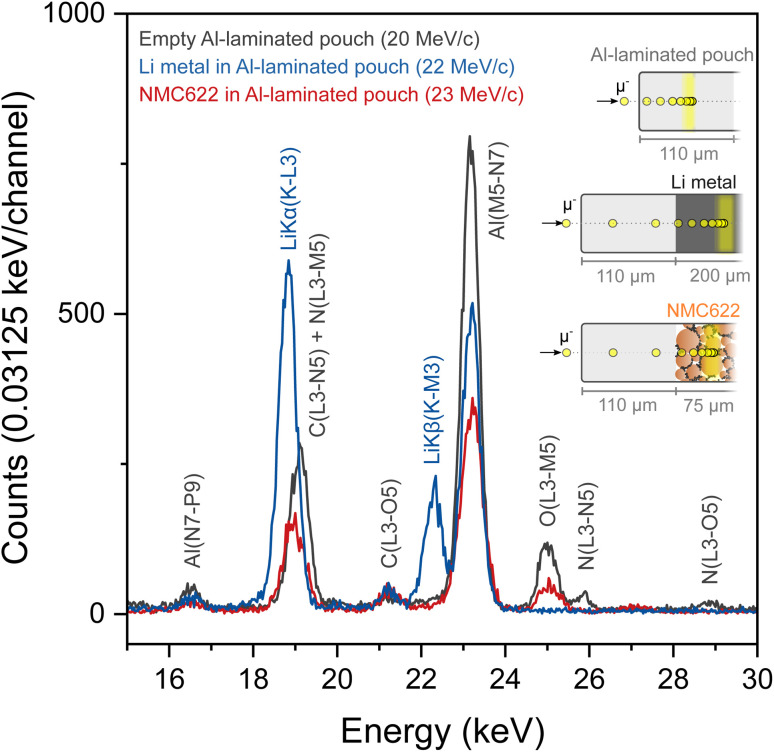
MIXE spectra narrowed in the Li K region from a reference laminated Al pouch, Li metal foil, and a lithiated NMC622 electrode. The Li peaks are marked in blue, while those from the laminated Al pouch are marked in grey.

Conveniently, MIXE produces muonic X-rays which are approximately two orders of magnitude more energetic than electronic X-rays, thereby considerably enhancing the distance that X-rays can travel before being absorbed by a material. To verify that Li muonic X-rays have a long escape depth and to test the efficiency of our system to detect them, the MIXE spectrum of Li metal foil placed in a laminated Al pouch (in blue) is overlapped as shown in Fig. 2 with that of an empty laminated Al pouch (in grey).

The empty laminated Al pouch, measured as a reference, displays X-ray transitions assigned to Al, C, N, and O, which is consistent with the layered structure of the pouch consisting of polyamide, aluminum, and polypropylene layers held together by polyester–polyurethane adhesives. The Li metal foil spectrum clearly features two peaks corresponding to Li K_α_ and Li K_β_ muonic X-rays at 18.8 keV and 22.3 keV respectively and two smaller peaks assigned to Al and C. The muonic X-rays generated in the pouch materials are absent from the Li metal spectrum indicating that the muons are successfully implanted in the Li metal layer and not at other depths in the sample. The strong intensity of the Li peaks confirms that the induced Li muonic X-rays were able to escape the pouch (with a thickness of 110 μm) and reach the array of detectors. Fig. 2 also shows that the Li K_α_ peak falls in the same region as a C and a N peak, albeit with slightly different centroids, which means that the analysis of layers whose composition contains these three elements will be more complex. The Li K_β_ peak is well separated from the Al peak.

The detectability of Li in a battery electrode using MIXE was assessed on a lithiated LiNi_0.6_Mn_0.2_Co_0.2_O_2_ (NMC622) electrode, vacuum-sealed in a laminated Al pouch (in red in Fig. 2). In the 15–30 keV range, peaks for Al, C, and O can be clearly observed. The detection of Li is more ambiguous because of the overlap between the Li K_α_ peak and the C peak. The Li K_β_ peak is not visible in this spectrum. The absence of Li peaks in the fully lithiated NMC sample, which is composed at 25 at% by Li atoms, highlights an important fundamental aspect of muon capture in heteroatomic structures: different elements have different probabilities for muon capture.^15^ Although muon capture probabilities do not directly scale with atomic numbers,^25^ heavier atoms tend to have higher capture probabilities than lighter ones: for instance, Ni has an approximately 16 times higher muon capture probability than Li (average capture probability of 2.88 ± 0.22 and 0.18 ± 0.04 relative to oxygen, respectively).^25^ Thus, the presence of heavy transition metal atoms in a structure can significantly compromise the chances of muons being captured by neighboring Li atoms. In other words, once implanted in an NMC electrode, negative muons are disproportionately captured by Ni, Mn, and Co, at the expense of Li, thereby dwarfing the Li muonic X-ray lines. In that respect, the detection of Na in electrodes employed in Na-ion batteries should be more favorable by virtue of its higher muon capture probability, which is approximately 5 times that of Li (1.00 ± 0.04, relative to oxygen). Regarding lithium, augmenting conventional high-purity germanium detectors with silicon drift detectors, whose resolution is high at low energies, might help detect the few muonic X-rays generated by Li inside transition metal containing cathode compounds. A strategy to monitor relative changes in the Li concentration in carbon-containing samples consists in calculating the ratio *r* = *I*_Li(K–L3)+C(L3–N5)_/*I*_C(K–L)_, where *I*_Li(K–L3)+C(L3–N5)_ is the combined intensity of the Li(K–L3) and C(L3–N5) lines between 18 and 20 keV and *I*_C(K–L)_ is the intensity of the C(K–L) line at 75 keV.^8^ As the ratio of the two C lines should be constant, any variations in *r* can be attributed to a change in the Li concentration.

### 4.2 Quantification of elemental ratios in NMC electrodes

Relative peak intensities in a muonic X-ray spectrum depend on a combination of experimental factors and quantum principles. For example, the energy-dependent efficiency of the germanium detectors is an experimental parameter, and the element-dependent muon capture probabilities are fundamental quantum properties affecting the relative intensity of peaks. The normalization of raw MIXE data to obtain the correct relative elemental ratios of a material has been thoroughly described in one of our previous publications.^11^

**Fig. 3:**
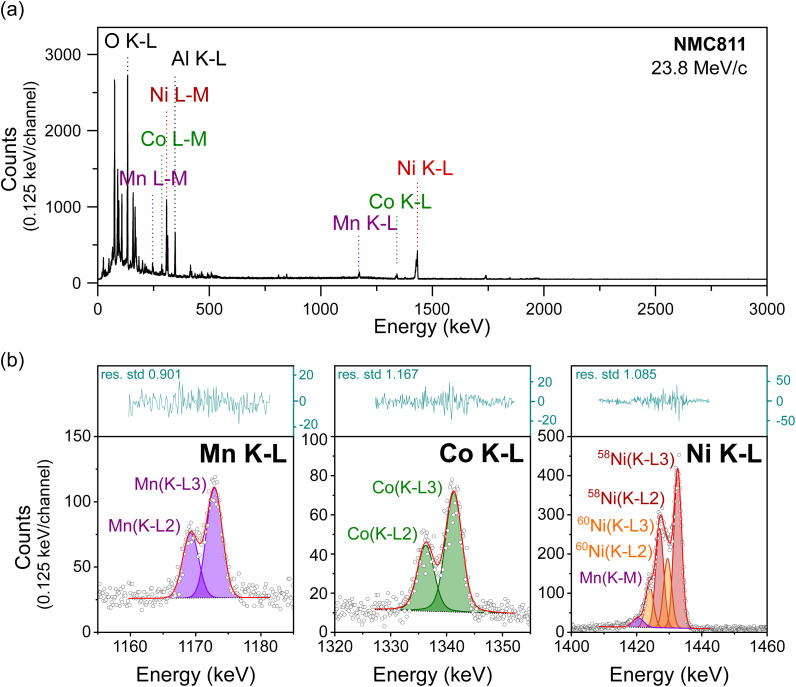
MIXE spectrum of an NMC811 electrode, (a) full muonic X-ray range measured, and (b) magnification of the Mn, Co, and Ni K–L regions whose fitted areas were used to calculate the transition metal ratio in the electrode. X-ray counts shown as circles; the linear background as a dotted line; μX-ray lines as coloured areas; the fitting envelope as a solid red line; residues as a blue line in the separate top box.

To confirm that MIXE provides accurate elemental quantification of a layer, we analyzed three samples from the family of NMC cathode materials, with different Ni : Mn : Co ratios, (i) NMC111, (ii) NMC622, and (iii) NMC811 as listed in Table 1. The full-range MIXE spectrum of the reference NMC811 electrode is presented in Fig. 3(a), with an identification of the K–L and L–M muonic X-ray lines of Ni, Mn and Co, and the K–L lines of O and Al (the spectra for NMC622 and NMC111 are shown in Fig. S2 and S3†). To calculate the relative ratios of Ni, Mn, and Co, their K–L peak areas were integrated and normalized by the detector efficiency at their respective energies. Fig. 3(b) shows the results of peak-fitting for the three transition metals K–L lines. Fig. 3(b) shows the capability of our system to clearly distinguish the K–L3 and K–L2 lines of these transition metals and, remarkably, to also distinguish between different isotopes in the case of Ni. Here, the fitting model considers peaks for the two most abundant isotopes of Ni, ^58^Ni and ^60^Ni. By taking the ratio of their K–L3 peak areas, the fraction of ^58^Ni to ^60^Ni is found to be 70 : 30 in the electrode, closely matching the natural ratio of these isotopes 72 : 28 (natural abundances of 68.1% and 26.2% respectively, and the remaining 5.7% being ^61^Ni, ^62^Ni, and ^64^Ni).^26^

**Table 1:** Experimentally measured ratios for three different NMC electrodes

NMC type	Nominal composition	Ni/(Ni + Mn + Co)	Mn/(Ni + Mn + Co)	Co/(Ni + Mn + Co)
NMC111	Li_1−*x*_Ni_0.33_Mn_0.33_Co_0.33_O_2_	0.32 ± 0.02	0.35 ± 0.02	0.32 ± 0.01
NMC622	Li_1−*x*_Ni_0.6_Mn_0.2_Co_0.2_O_2_	0.58 ± 0.05	0.21 ± 0.02	0.21 ± 0.02
NMC811	Li_1−*x*_Ni_0.8_Mn_0.1_Co_0.1_O_2_	0.80 ± 0.03	0.103 ± 0.005	0.10 ± 0.02

The measured ratios of the three electrodes are presented in Table 1. The results clearly indicate the accuracy of the technique and of the normalization procedure to quantify the composition of each electrode. The following section will also demonstrate that quantification is accurate across a range of muon implantation depths and is independent of muon momentum.

### 4.3 Depth profiling of an NMC811/graphite pouch cell and muon implantation accuracy

**Fig. 4:**
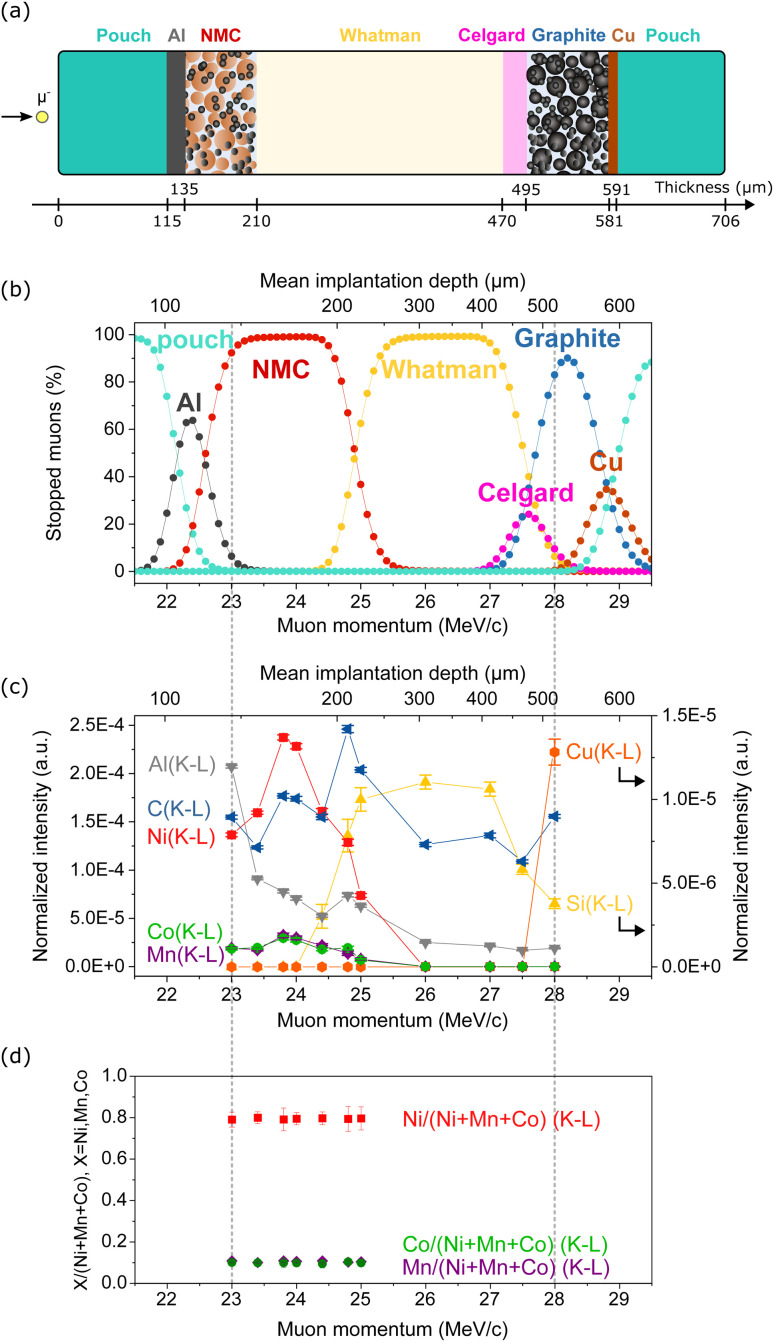
(a) Schematic of the analyzed NMC‖graphite cell. (b) Fraction of muons predicted to stop in the different layers of the cell from stopping profile simulations. (c) MIXE depth-profile showing the normalized intensities of selected (K–L) lines. Solid lines connecting data points are used as a guide to the eye and have no physical signification. (d) Quantification of the transition metal ratios in the NMC electrode.

To validate the depth-profiling capability of MIXE for battery applications and to test the muon implantation accuracy of our system, we analyzed an NMC811/graphite pouch cell, schematically represented in Fig. 4(a).

Before conducting the experiment, simulations were performed using the parameters listed in Table S1† to predict muon implantation profiles within the pouch cell. Nine of these simulated profiles are shown in Fig. S4,† revealing that the muon stopping profile is highly influenced by the density of each layer. For each profile, along with an additional 112 simulations spanning a momentum range of 18 MeV/c to 30 MeV/c in 0.1 MeV/c intervals, the mean muon-implantation depth and standard deviation were calculated, as displayed in Fig. S5.† These results also show a larger spread in implantation depths within low-density layers, such as the Whatman layer, reflected in the higher standard deviations. For ease of comparison with experimental data, the simulation results are presented in a third format in Fig. 4(b), which depicts the fraction of muons stopping in each pouch cell layer as a function of muon momentum. Additionally, the simulated mean muon-implantation depth is provided on a secondary *x*-axis in Fig. 4(b) to facilitate direct comparison with Fig. 4(a).

The outcome of the simulation was the selection of muon momenta allowing eleven depths across the cell (five in the NMC electrode, three in the glass-fiber separator, one in the Celgard separator, and two in the graphite electrode) to be probed as depicted in Fig. 4(a). MIXE spectra were acquired for 30 minutes at each depth. Three of these eleven MIXE spectra, acquired at muon momenta of *p* = 24.0 MeV/c, *p* = 26.0 MeV/c and *p* = 28.0 MeV/c, are presented as examples in Fig. S6.† The identification of all the peaks in Fig. S6† demonstrates the remarkable fingerprinting nature of MIXE. For each element, the muon cascade from levels *n*_μ_ ≈ 14 to *n*_μ_ = 1 results in multiple X-ray emissions, many of which are detected by our system. Twelve μX-ray transitions are observed for Ni in Fig. S6† in the spectrum acquired at *p* = 24.0 MeV/c, including N series transitions such as Ni(N7–Q9) (7g_9/2_–4f_7/2_ in spectroscopic notation) transition around 92.85 keV.

After peak identification, the MIXE spectra acquired at the eleven depths were individually fitted and normalized as described in the Methods section. The normalized integrated areas of K–L transitions of the main elements composing the cell are used in Fig. 4(c) to demonstrate the depth-profiling capability of MIXE. The Ni, Co and Mn (K–L) lines are signatures of the positive electrode, the Si(K–L) line is used as a signature for the glass-fiber separator, and the Cu(K–L) line comes from the current collector of the graphite electrode. The C and Al(K–L) lines are also included in Fig. 4(c) but are less precise descriptors of an individual layer; indeed, carbon is present throughout the cell as (1) graphite in the negative electrode, (2) a conductive additive in both electrodes, (3) in the polymeric binders in both electrodes, (4) in the polypropylene separator, and (5) in the electrolyte solvents. The aluminium signal originates either from the current collector on the positive electrode or as a parasitic signal from the laminated Al pouch and from the experimental apparatus if muons are not penetrating the sample at the intended spot. Fig. 4(b) and (c) show that the experimental results follow the simulation prediction and that muons can accurately be implanted in the cell. The maximum intensity of the Ni, Co and Mn(K–L) lines is observed at a muon momentum of 23.8 MeV/c which corresponds well with the results of the simulation. From 24.4 MeV/c onwards, the Si(K–L) line can be observed, indicating that muons are implanting in the glass-fiber separator.

The detection of Cu in the MIXE spectrum acquired at 28 MeV/c (see Fig. S6†) confirms the predictions of the simulation and shows that muons can be implanted in the negative electrode current collector. This experiment demonstrates that a depth-profile of a full pouch cell with a thickness of ∼700 μm is possible with MIXE, in good agreement with the results from simulations.

The ratios of transition metals in the NMC electrode were calculated and are presented in Fig. 4(d) for all the momenta for which Ni, Mn and Co lines were detected. Following up from Section 4.2, we can again observe that a Ni : Co : Mn ratio of ∼8 : 1 : 1 is measured across the entire NMC electrode. These results are another confirmation of the quantitative nature of MIXE and demonstrate that the quantification accuracy is independent of the depth of muon implantation.

## Conclusions

This article serves as an introduction to MIXE for the battery research community, highlighting its advantages as a novel non-destructive cell characterization method with high probing depth. Benefiting from the high intensity of the PSI muon beamline and its custom detection system, our experiments have demonstrated that MIXE can analyze changes in the chemical composition of battery layers over a depth range of 10^1^–10^3^ μm, far exceeding the depth resolution of other X-ray characterization techniques. In our experiments, depth profiles of NMC‖graphite full cells were acquired. Muons were accurately implanted at selected depths by tuning their momentum according to the results of simulations. Our experiments confirm the quantitative nature of MIXE and its ability to detect nearly all elements of the periodic table, including light ones, such as Li.

The MIXE technique still requires further development to address some of its current limitations, which is the focus of our ongoing work. Currently, the beam spot size is a circular area with a diameter of a few centimeters. The addition of a muon tracking chamber will enhance the determination of the entry point of each implanted muon in a sample, advancing MIXE towards becoming a tomographic technique. Utilizing this muon tracking chamber alongside a continuous muon source, where muons are implanted one at a time with roughly ten muon lifetimes between each implantation, will also yield more precise muonic X-ray spectra: this improvement is achieved by retaining only the X-rays produced after a muon penetrates the region of interest through post-processing. Future developments will also need to explore the use of multiple detector types. This approach could, for example, enhance the detection of lithium, which is feasible in metallic form with HPGe detectors but more challenging in compounds such as electrodes. For sodium batteries, detecting sodium should be less problematic due to its higher muon capture probability.

In conclusion, MIXE's distinctive advantage over other spectroscopic X-ray techniques is its extended analysis depth. MIXE should be considered in experiments where the long-range mobility of ions in batteries needs to be investigated. To only name a few, MIXE could bring answers to questions related to heterogeneous (dis)charge kinetics across the thickness of high mass-loading electrodes, corrosion/dissolution of layers and subsequent poisoning of other layers in a cell, or the detection of alkali metal filaments (dendrites) forming in solid electrolytes in all-solid-state batteries.

## Data availability

All the datasets generated for this article including fittings are available on Zenodo (DOI: https://doi.org/10.5281/zenodo.12188474).

## Author contributions

EQ: conceptualization, investigation, formal analysis, visualization, writing – original draft. SB: conceptualization, investigation, formal analysis, methodology, software, data curation, writing – original draft. MH: formal analysis, writing – review & editing. LG: conceptualization, investigation, methodology, software, data curation. QW: investigation, writing – review & editing. RA: conceptualization, investigation. GM: investigation.SA: investigation. IC: investigation. DD: investigation. ZG: investigation. FH: investigation. GJ: investigation. AK: investigation. HL: investigation. CM: investigation. KN: investigation. MN: investigation. TP: investigation. AS: investigation. KvS: investigation. TS: investigation. SMV: investigation. CV: investigation. CW: investigation. CB: funding acquisition, writing – review & editing. AA: investigation, funding acquisition, project administration, writing – review & editing. AR: investigation, funding acquisition, project administration, writing – review & editing.

## Conflicts of interest

There are no conflicts to declare.

## Supplementary Material

TA-013-D4TA05112B-s001
