# Supplementary material for: Overcoming the probing-depth dilemma in spectroscopic analyses of batteries with muon-induced X-ray emission (MIXE)
Source: J Mater Chem A Mater. 2024 Dec 4;13(3):2275–84. doi: 10.1039/d4ta05112b (PMC11639054; doi:10.1039/d4ta05112b)
Supplement: TA-013-D4TA05112B-s001 [file TA-013-D4TA05112B-s001.pdf]

Supplementary Information (SI) for Journal of Materials Chemistry A.
This journal is © The Royal Society of Chemistry 2024

Overcoming the probing-depth dilemma in spectroscopic analyses of batteries with muon-induced X-ray emission (MIXE)

Edouard Quérel[1,*], Sayani Biswas[2,*,†], Michael Heiss[2], Lars Gerchow[2], Qing Wang[1], Ryo Asakura[1], Gian Müller[1], Debarchan Das[2], Zurab Guguchia[2], Fabian Hotz[2], Gianluca Janka[2], Andreas Knecht[2], Hubertus Luetkens[2], Charles Mielke III[2], Carlos Vigo[2], Chennan Wang[2], Katharina von Schoeler[2,3], Stergiani Marina Vogiatzi[2,3], Toni Shiroka[2], Thomas Prokscha[2], Shunsuke Asari[4], I-Huan Chiu[4], Akira Sato[4], Kazuhiko Ninomiya[5], Megumi Niikura[6], Corsin Battaglia[1,7,8], Alex Amato[2], Arndt Remhof[1]

[1] Empa, Swiss Federal Laboratories for Materials Science and Technology, 8600 Dübendorf, Switzerland

[2] Center for Neutron and Muon Sciences, Paul Scherrer Institute, 5232 Villigen PSI, Switzerland

[3] Institute for Particle Physics and Astrophysics, ETH Zürich, 8093 Zürich, Switzerland

[4] Graduate School of Science, Osaka University, 1-1 Machikaneyama, Toyonaka 560-0043, Osaka, Japan

[5] Institute of Radiation Sciences, Osaka University, Toyonaka, Osaka, Japan

[6] RIKEN Nishina Center for Accelerator-Based Science, 2-1 Hirosawa, Wako 351-0198 Saitama, Japan

[7] Department of Information Technology and Electrical Engineering, ETH Zürich, 8092 Zürich, Switzerland

[8] Institute of Materials, School of Engineering, EPFL, 1015 Lausanne, Switzerland

December 9, 2024

*Correspondence: Edouard.Querel@empa.ch and sayani.biswas@stfc.ac.uk

†Current affiliation: ISIS facility, STFC Rutherford Appleton Laboratory, Didcot OX11 OQX, United Kingdom

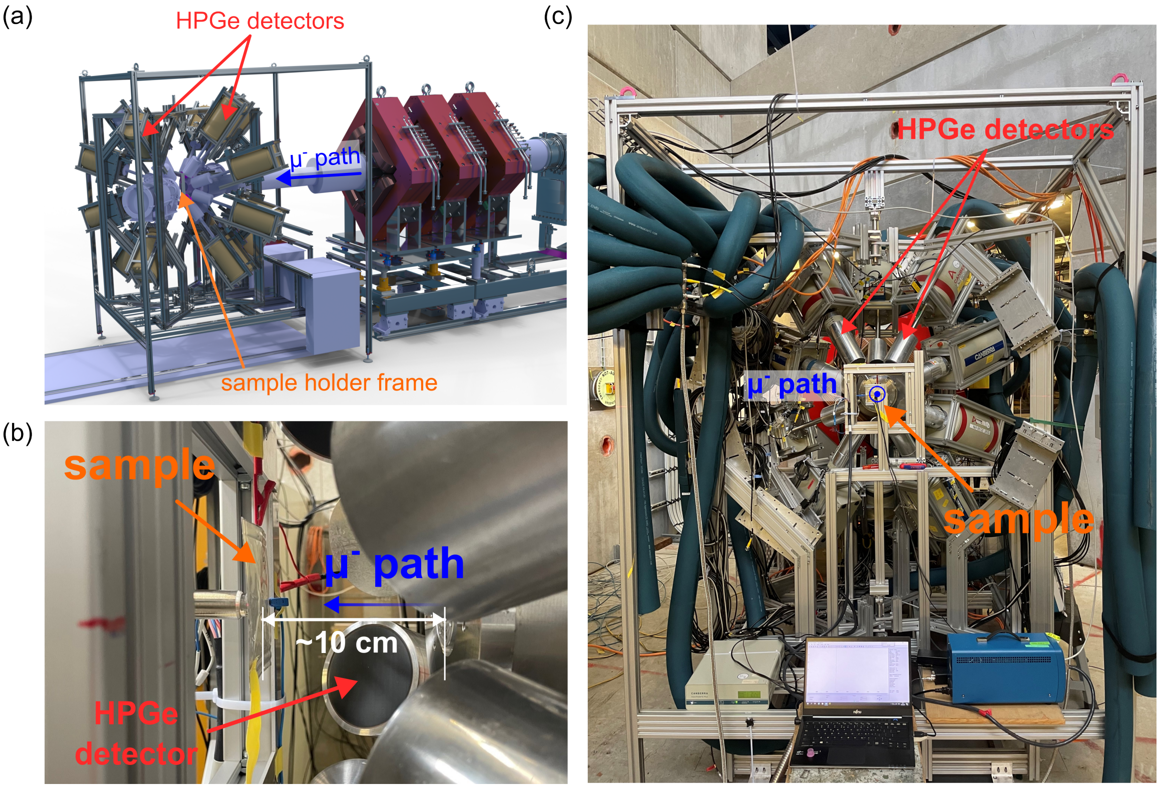

Figure 1: (a) CAD models of the MIXE setup at PSI showing the μ^- path after exiting the last set of quadrupole magnets and the detection frame with HPGe detectors arranged radially around the sample (reproduced with permission)[1], (b) Side-view photograph showing the sample attached in its frame, placed in air, at a distance of $\sim$10 cm from the window from which μ^- exit the accelerator, (c) Photograph of the detection setup coaxial to the μ^- path (μ^- come towards the reader).

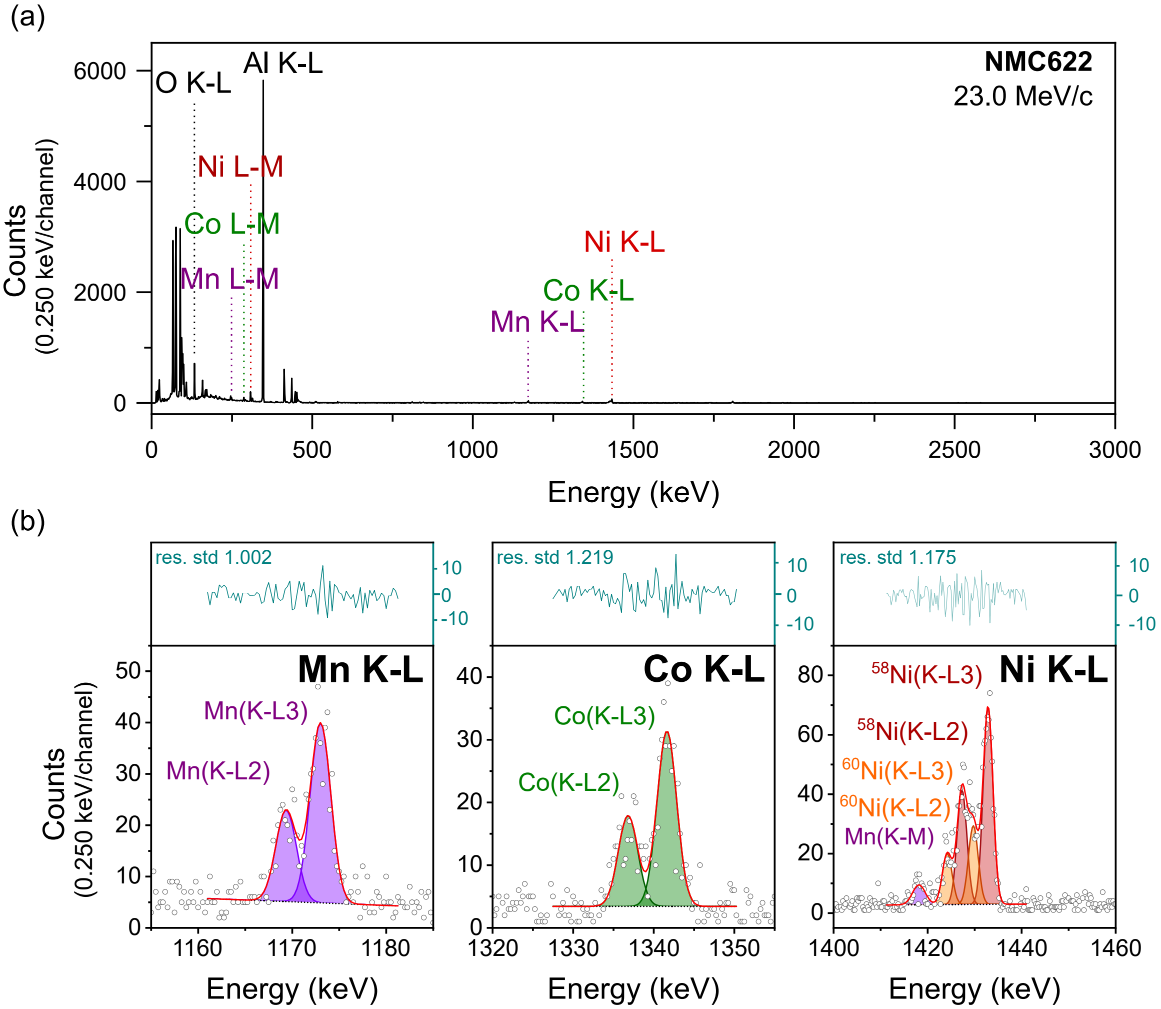

Figure 2: Fitting of the Mn, Co, and Ni K-L regions of a reference NMC622 electrode

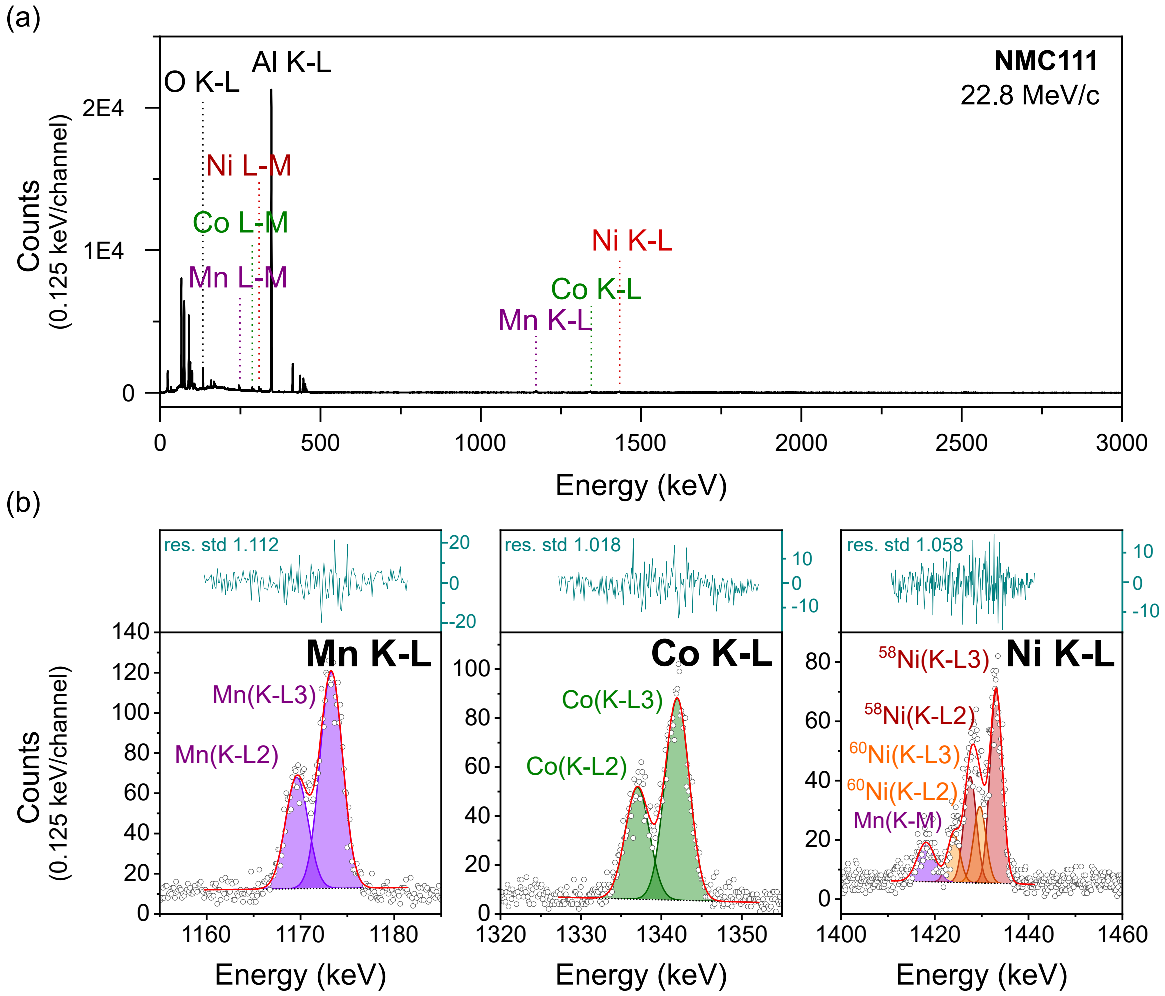

Figure 3: Fitting of the Mn, Co, and Ni K-L regions of a reference NMC111 electrode

**Layer**	**Composition**	**Density (g/cm^3)**	**Thickness (μm)**	**Porosity (%)**	**Corrected density (g/cm^3)**
Pouch	Polyamide	1.1	30	0	
	Al	2.7	40	0	
	Polypropylene	0.9	45	0	
Al	Al	2.7	0	0	
NMC	NMC811	4.77	75	39	3.38
	LP57	1.20			
Whatman	SiO_2(90%)/B_2O_3(10%)	2.65/2.46	260	93	1.30
	LP57	1.20			
Celgard	Polypropylene	0.45	25	55	1.05
	LP57	1.20			
Graphite	Graphite	2.26	86	37	1.87
	LP57	1.20			
Cu	Cu	8.96	10	0	

Table 1: List of parameters used in the simulations for Figure 4. Corrected densities are only calculated in the case of a porous layer impregnated by the electrolyte. For the electrode layers, the density of the active material was taken to simplify the calculations. NMC811: $LiNi_{0.8}Mn_{0.1}Co_{0.1}O_2$. LP57: 1M $LiPF_6$ in EC:EMC 3:7. EC: ethylene carbonate, EMC: ethyl methyl carbonate.

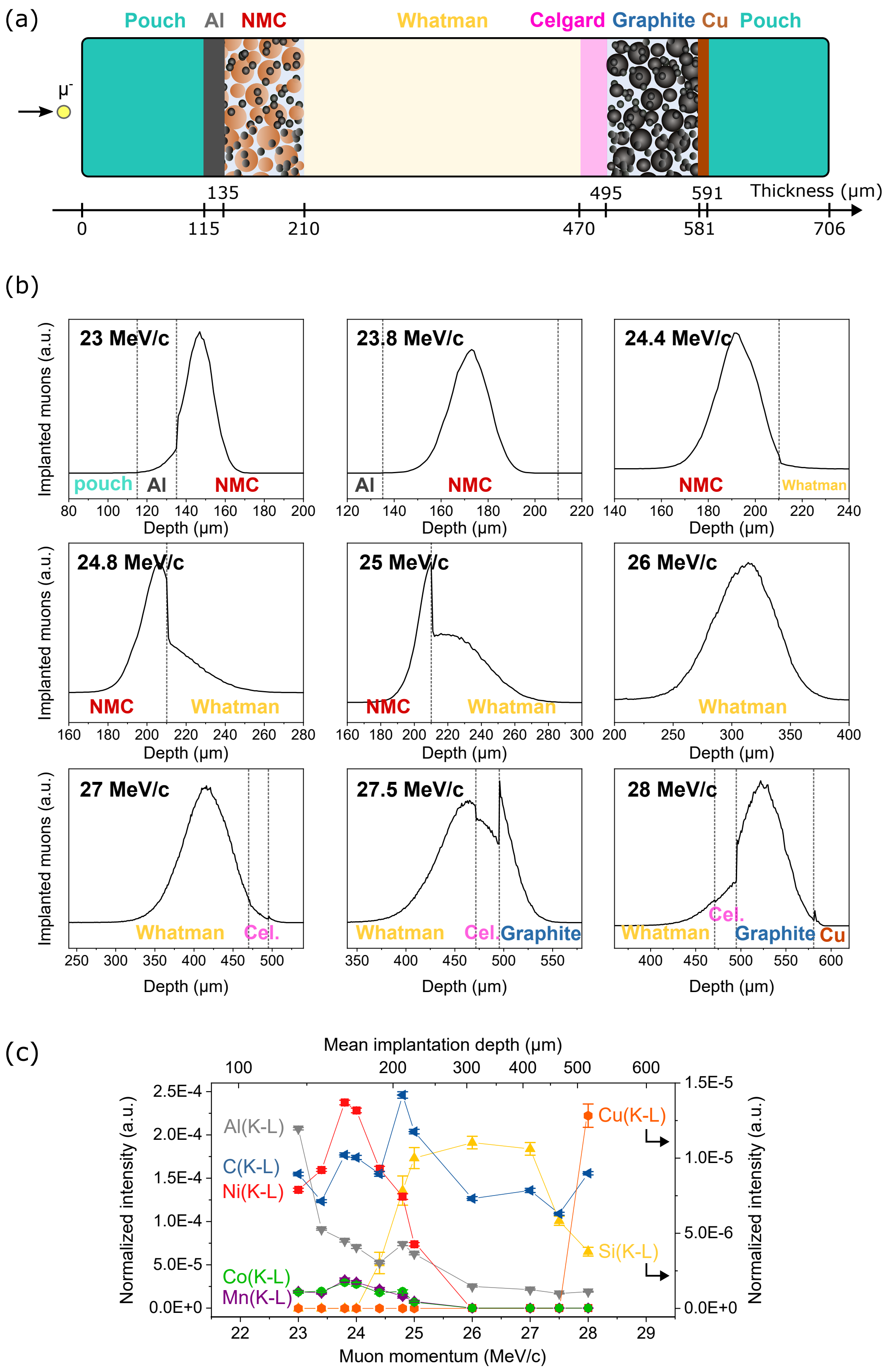

Figure 4: (a) Schematic of the cell; (b) Muon implantation profiles at selected momenta used in experiment; (c) Experimental results (same as the one presented in Figure 4, reproduced here for easy comparison with the implantation profiles)

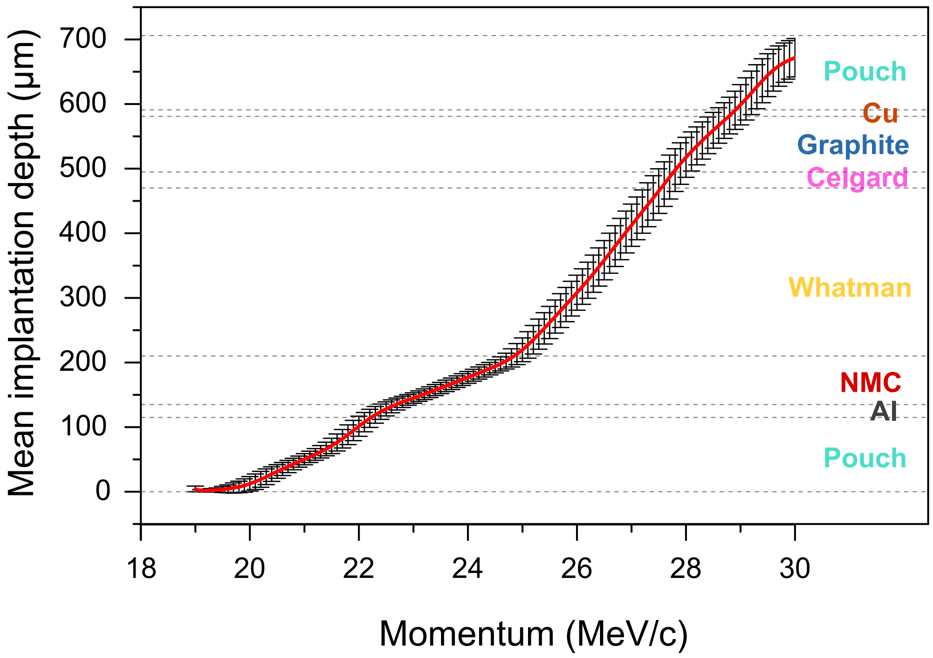

Figure 5: Simulated mean muon implantation depth as a function of momentum. Error bars represent the standard deviation at each muon momentum.

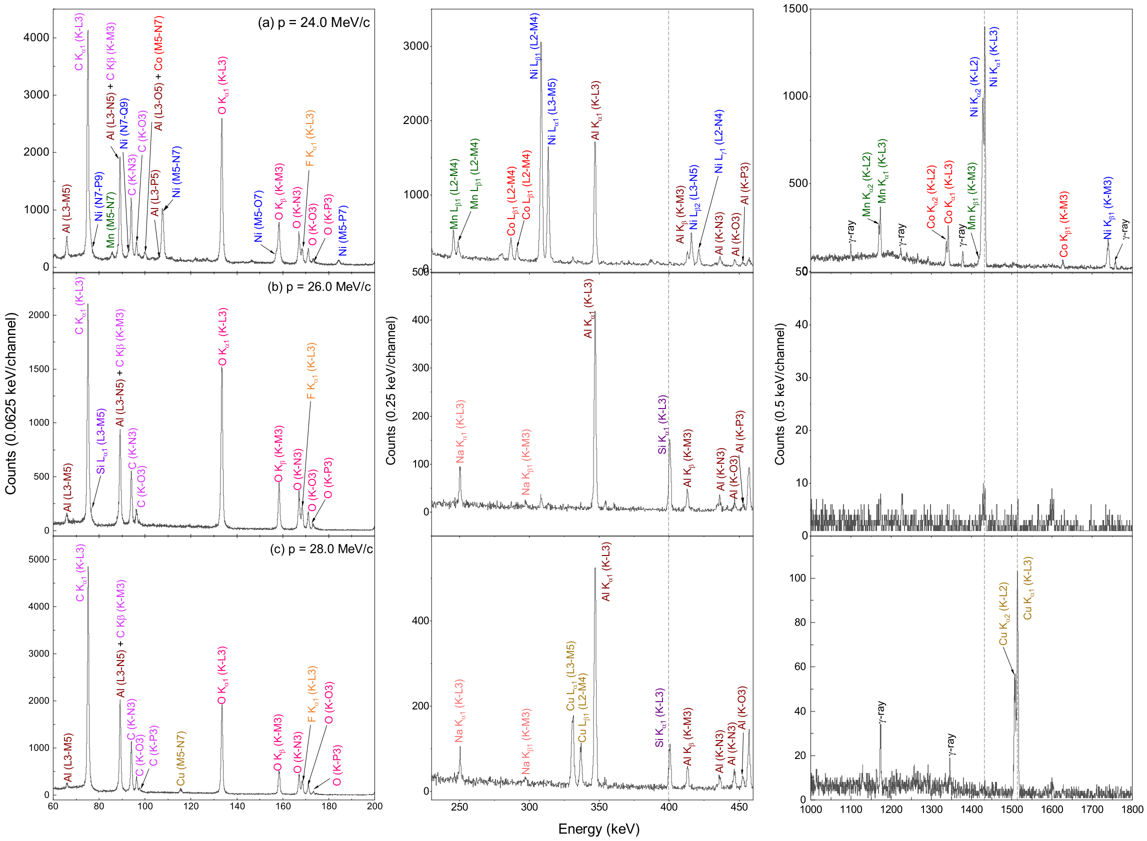

Figure 6: Muonic X-ray spectra for the NMC811/graphite cell at three different muon momenta (a) p = 24.0 MeV/c, (b) p = 26.0 MeV/c, and (c) p = 28.0 MeV/c, with the energy ranges 60 - 200 keV (left column), 230 - 460 keV (middle column) and 1000 - 1800 keV (right column), respectively. Dashed lines for the Si, Ni and Cu (K-L) lines at 400, 1432 and 1514 keV, respectively, are drawn.

References

[1] L. Gerchow *et al.*, Germanium array for non-destructive testing (giant) setup for muon-induced x-ray emission (mixe) at the paul scherrer institute, Review of Scientific Instruments **94** (2023).
